# Updated Taxonomy of Chinese *Craterellus* (Hydnaceae, Cantharellales) with Three New Species Described

**DOI:** 10.3390/life15020157

**Published:** 2025-01-23

**Authors:** Tian Jiang, Lei Zhao, Xu Zhang, Hua-Zhi Qin, Hui Deng, Xiao-Dong Mu, Nian-Kai Zeng

**Affiliations:** 1 School of Pharmacy, Hainan Medical University, Haikou 571199, China; jiangtian7777@hainmc.edu.cn (T.J.); huazhiqin2022@163.com (H.-Z.Q.); denghui4718@hainmc.edu.cn (H.D.); 2 Ministry of Education Key Laboratory for Ecology of Tropical Islands, Key Laboratory of Tropical Animal and Plant Ecology of Hainan Province, College of Life Sciences, Hainan Normal University, Haikou 571158, China; 17786939546@163.com; 3 Hainan Smart Rainforest Center, Haikou 570203, China; 15733255449@163.com; 4 Hainan Research Academy of Environmental Sciences, Haikou 571126, China

**Keywords:** molecular phylogeny, morphology, new taxa, taxonomy, trumpet mushroom

## Abstract

Species of *Craterellus* are interesting and important due to their mycorrhizal properties, medicinal value, and edibility. Despite extensive research on *Craterellus* in China, its taxonomy remains inadequately understood. This study presents three newly described species of *Craterellus*, namely *C. albimarginatus*, *C. involutus*, and *C. longitipes*, identified through morphological and phylogenetic analyses, with the goal of refining the taxonomy of Chinese *Craterellus*.

## 1. Introduction

*Craterellus* Pers. (Hydnaceae, Cantharellales), with *C. cornucopioides* (L.) Pers. as the type species, is distinguished by its small, funnel-shaped basidioma and hollow stem [1]. It is widely distributed in the temperate regions of the northern hemisphere and is frequently found in the tropics [2]. Ecologically, *Craterellus* usually forms ectomycorrhizal relationships with various host plants including species from Dipterocarpaceae, Fagaceae, Malvaceae, Myrtaceae, Pinaceae, and Salicaceae, contributing significantly to the biodiversity of forest ecosystems [3,4]. Furthermore, species of *Craterellus* are edible and medicinal, and they contain chemical components with pharmacological effects such as immunomodulatory, anti-inflammatory, and anti-mutagenic properties [5,6,7,8,9].

The genus *Craterellus* exhibits high species diversity, with approximately 173 recognized species (source: http://www.indexfungorum.org, accessed 10 December 2024). However, taxonomic classification within the genus is complicated by subtle morphological differences among species [2]. To clarify the confusion in the taxonomy of *Craterellus* species, analyses of phylogeny and morphological characteristics were performed by mycologists [10,11,12,13,14,15,16,17]. Moreover, six well-supported subgenera, viz. *Cariosi* T. Cao & H. S. Yuan, *Craterellus*, *Imperforati* T. Cao & H. S. Yuan, *Lamelles* T. Cao & H. S. Yuan, *Longibasidiosi* T. Cao & H. S. Yuan, and *Ovoidei* T. Cao & H. S. Yuan, were revealed [10].

In China, species of *Craterellus* have also attracted the attention of mycologists, and eighteen taxa of the genus have been identified or described [6,8,9,10,11,12,13,14,15,16,17]. However, the diversity of the genus still remains incompletely understood. In one previous study, three new species of this genus from tropical–subtropical regions of China were described by our research team [16]. Recently, new collections of *Craterellus* were made and studied using both morphological and molecular phylogenetic analyses. A multilocus phylogeny for *Craterellus* was constructed based on a combined dataset of 28S, ITS, and *TEF*1, aiming to update the taxonomy of *Craterellus* in China.

## 2. Materials and Methods

### 2.1. Morphological Studies

Digital photographs and field notes were systematically recorded from fresh basidiomata in the field, documenting their sizes, colorations, any color changes, and the associated symbiotic plant species. Specimens were subjected to drying at 50–60 °C [18], subsequently stored in a refrigerator at minus 20 degrees for two weeks, and finally deposited in the Hainan Biodiversity Science and Technology Museum (FHMU), Hainan Province of China. Color codes were referenced from Wanscher and Kornerup [19]. Sections of the pileipellis were excised from the pileus between the central and marginal areas and subsequently mounted in a 5% potassium hydroxide (KOH) solution; 1% Congo Red staining was used as an adjunct for observation. The specimens were then examined and measured using a bright-field microscope (CX23, Olympus, Tokyo, Japan). The notation is indicated as “n” basidiospores measured from “m” basidiomata of “p” collections [n/m/p]. The basidiospore dimensions are given as (a–)b–e–c(–d), where “b–c” denotes at least 90% of the measured values (5th to 95th percentile), and extreme values (a and d) are shown in parentheses whenever they occur (a < 5th percentile, d > 95th percentile); “e” stands for the average length/width of the basidiospores. The length/width ratio of basidiospores is denoted by “Q”, and the average “Q” of these basidiospores, along with its standard deviation, is denoted by “Qm”. The words that describe a basidioma’s size are derived from Bas [20].

### 2.2. Molecular Procedures

To obtain materials for DNA extraction from the fresh basidiomata, a small piece of the pileus was excised, subsequently wrapped in paper, and finally placed in a sealed bag with silica gel. Total genomic DNA was extracted from 10 to 20 mg of dried basidiomata using the Magnetic Beads Genomic DNA Extraction Kit (Magen, Guangzhou, China), following the manufacturer’s protocol. After extraction, 2 µL of the DNA sample was aliquoted, and its concentration and purity were assessed using a NanoDrop 8000 spectrophotometer (Thermo Fisher Scientific, Waltham, MA, USA). To check DNA integrity, 2 µL of the DNA sample was mixed with 2 µL of bromophenol blue loading dye and loaded onto a 1% agarose gel in TAE buffer for electrophoresis. The gel was run at a constant voltage of 100 V for (20 min). Negative controls were included in each batch of DNA extractions to rule out contamination.

Large subunit ribosomal DNA (28S), the nuclear ribosomal internal transcribed spacer (ITS), and translation elongation factor 1-α (*TEF*1) gene fragments were amplified by PCR using the universal primer pairs ITS5/ITS4 [21], LR0R/LR5 [22,23], and tefF/tefR [24], respectively. PCR procedures were conducted according to the methods outlined by An et al. [25] and Zhang et al. [26]. The PCR reaction was performed in a 30 µL mixture containing 1 µL of DNA template (approximately 20 ng), 2 µL of each forward and reverse primer (5 pmol/µL), 15 µL of 2 × Taq PCR Master Mix, and 10 µL of ddH_2_O. The amplification program included an initial denaturation at 95 °C for 5 min, followed by 35 cycles of 95 °C for 30 s, 50 °C for 30 s (annealing), and 72 °C for 1 min (extension). PCR products were verified by electrophoresis on a 1% (*w*/*v*) agarose gel.

The PCR products were sequenced using an ABI 3730xL DNA Analyzer (Huayu Gene, Wuhan, China). Forward and reverse sequences were assembled using BioEdit v7.0.9 [27]. The successfully sequenced and assembled fragments were compared against the NCBI nt (nucleotide)/nr (non-redundant protein) databases using BLAST (Basic Local Alignment Search Tool) to identify closely related sequences. All newly acquired sequences were submitted to GenBank (Table 1).

### 2.3. Dataset Assembly

A total of 39 DNA sequences (15 from 28S, 11 from ITS, and 13 from *TEF*1) were newly generated from 24 collections in the present study (Table 1). These sequences were included in a concatenated dataset (28S, ITS, and *TEF*1) and cross-referenced with sequences from previous research and GenBank (Table 1). According to Zhang et al. [16], *Hydnum minus* Yanaga & N. Maek and *H. cremeoalbum* Liimat. & Niskanen were selected as the outgroup. A separate alignment of the sequences of 28S, ITS, and *TEF*1 regions was performed to check for phylogenetic disagreement. Phylogenetic tree diagrams, constructed using a single DNA sequence for each analysis, exhibited the same topologies, showing that there were no contradictions between the signals derived from the different gene segments. The alignment of the three datasets (28S, ITS, and *TEF*1) was performed using MUSCLE v3.6 [45], followed by concatenation using Phyutility v2.2 for subsequent analyses [46].

### 2.4. Phylogenetic Analyses

Methods of maximum likelihood (ML) and Bayesian inference (BI) were applied to analyze the integrated nuclear dataset. RAxML 7.2.6 was employed to construct the maximum likelihood tree and perform bootstrap (BS) analysis [47]. In the ML analysis, default parameter values were applied, except for the model, which was set to GTRGAMMA. Nonparametric bootstrapping with 1000 replicates was employed to derive statistical support. BI was carried out with the CIPRES Science Gateway portal using MrBayes v3.1 [48,49]. MrModeltest v2.3 was employed to identify the most appropriate models of nucleotide substitution for 28S (GTR + I + G), ITS (HKY + I + G), and *TEF*1 (SYM + G) according to the Akaike information criterion [50]. For the combined nuclear dataset (28S, ITS, and *TEF*1), Bayesian analysis was run for 30 million generations, with an average deviation of split frequencies of 0.004598. The initial 25 percent of sampled generations were discarded to account for burn-in, and the Bayesian posterior probabilities (PP) were estimated for the majority consensus tree based on the retained Bayesian trees.

## 3. Results

### 3.1. Molecular Data

**Figure 1:**
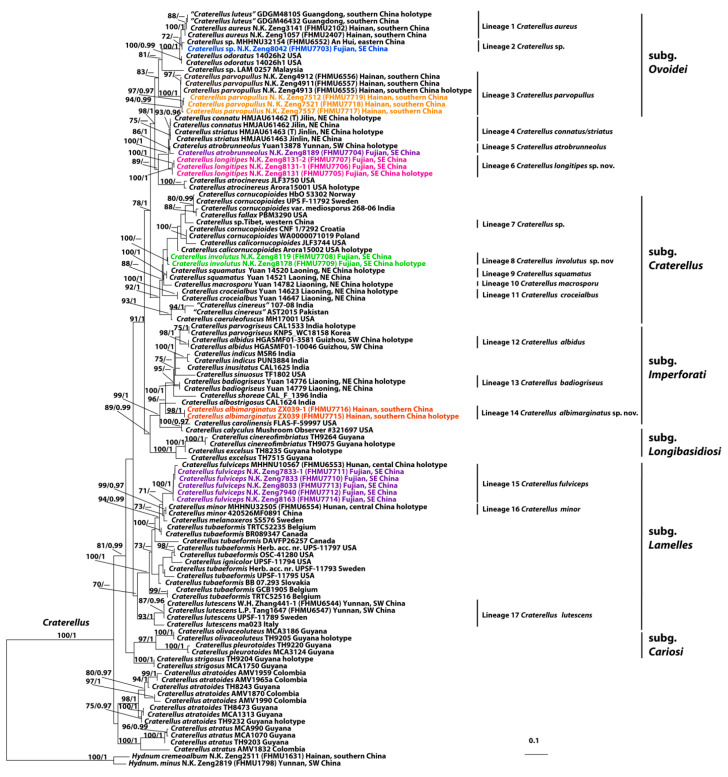
A maximum likelihood (ML) phylogenetic tree of *Craterellus* was derived from the combined dataset (28S + ITS + *TEF*1). ML bootstrap values (BS ≥ 70%) and Bayesian posterior probabilities (PP ≥ 0.95) are shown at the nodes of individual branches.

The dataset, combining 28S, ITS, and *TEF*1, included 109 sequences with 2644 nucleotide positions, with the alignment available in TreeBASE (31766) [https://treebase.org/treebase-web/home.html (accessed on 22 October 2024)]. The Bayesian analyses produced topologies identical to those of the ML analysis, with slight differences in statistical support (Figure 1). Based on the molecular data, sixteen independent lineages were identified within the Chinese species of *Craterellus* (Figure 1). Three new lineages were discovered in the current study (Lineages 6, 8, and 14). Lineage 6, with strong statistical support (BS = 100%, PP = 1.0), comprised three new collections (FHMU7705, FHMU7706, and FHMU7707) from southeastern China; in lineage 8, two collections (FHMU7708 and FHMU7709) also from southeastern China grouped together with 100% RAxML likelihood bootstrap and 0.8 posterior probability; in lineage 14, two collections (FHMU7715 and FHMU7716) from southern China were clustered together with high statistical support (BS = 98%, PP = 1.0).

### 3.2. Taxonomy

***Craterellus albimarginatus*** N.K. Zeng, T. Jiang & Xu Zhang, **sp. nov.**

MycoBank: MB 856885

**Figure 2:**
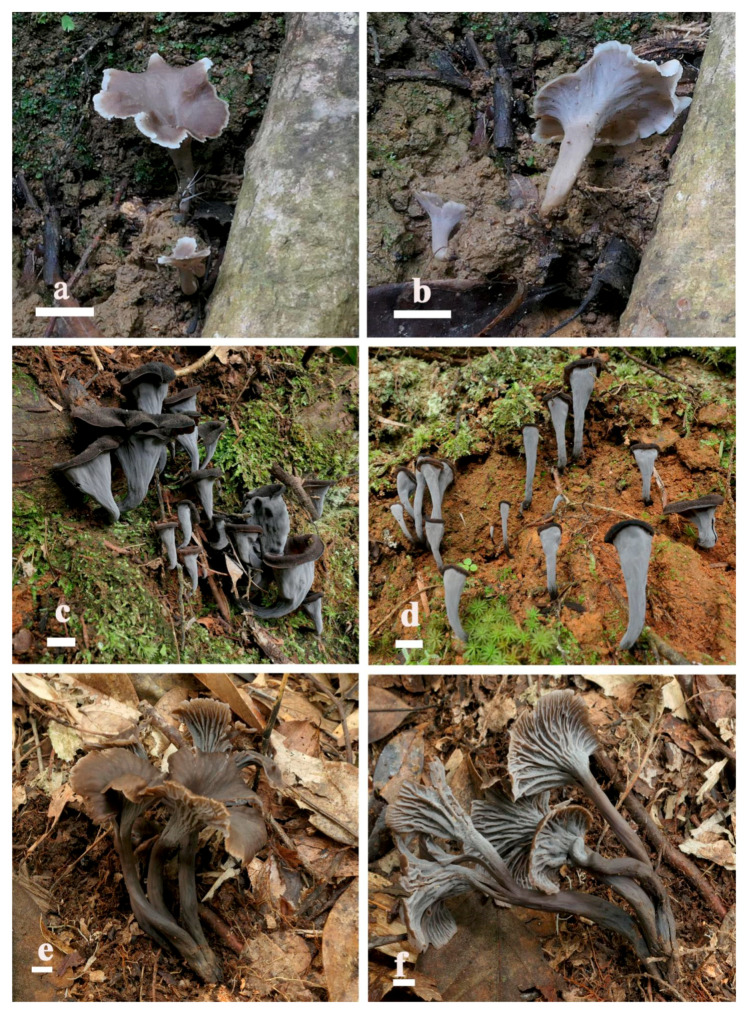
Basidiomata of *Craterellus* taxa. (**a**,**b**) *C. albimarginatus* (FHMU7715, holotype); (**c**,**d**) *C. involutus* (FHMU7709, holotype); (**e**,**f**) *C. longitipes* (FHMU7705, holotype). Scale bars: (**a**–**f**) = 1 cm. (**a**,**b**) photos by X. Zhang; (**c**–**f**) photos by N.K. Zeng.

**Figure 3:**
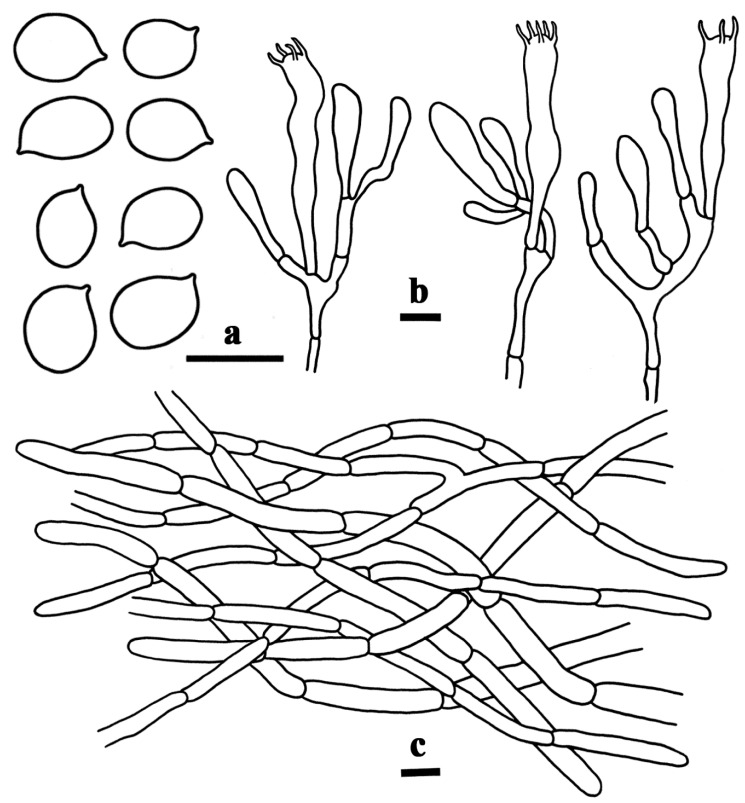
Microscopic structures of *Craterellus albimarginatus* (FHMU7715, holotype). (**a**) Basidiospores. (**b**) Basidia. (**c**) Pileipellis. Scale bars: (**a**–**c**) = 10 µm. Drawing by T. Jiang.

Figure 2a,b and Figure 3.

Etymology—Latin “*albi*-”, meaning white, and “*marginatus*”, meaning margin, refer to the white margin of the new species.

Holotype—CHINA. Hainan Province: Wuzhishan, Hainan Tropical Rainforest National Park, elev. 600 m, 9 June 2022, X. Zhang039 (FHMU7715). GenBank accession number: 28S = PQ604676, ITS = PQ611003.

Diagnosis—*Craterellus albimarginatus* is distinguished from the closest species of *Craterellus* by its very small basidioma, the distinct boundary between the pileus and stipe, a grayish-brown pileus with a pure white margin, a white hymenophore tinged with violet, smaller basidiospores, and its association with Fagaceae trees.

Description—Pileus 0.7–2.2 cm diameter, center strongly depressed, margin broadly wavy or unevenly folded; surface dry and slightly rough, grayish-brown (5D6), but margin pure white (1A1); context very thin, grayish white. Hymenophore slightly veined, spreading down; folds approximately 0.05 cm broad, white (1A1) tinged with violet (15A2). Stipe 0.7–1.5 × 0.1–0.4 cm, central, hollow, subcylindrical; surface dry, beige-almond (2B2); basal mycelium white (1A1). Odor indistinct. Spore print not obtained.

Basidiospores [40/2/2] 7–7.9–9(–9.5) × 5–5.6–6.5 µm, Q = (1.23–)1.27–1.60(–1.80), Qm = 1.41 ± 0.11, ellipsoid, rarely subglobose, smooth, inamyloid, marginally thickened walls (up to 0.5 µm), yellowish in KOH. Basidia 46–65 × 5–7 µm, 3–5-spored, subcylindrical, somewhat curving, marginally thickened walls (up to 0.5 µm), yellowish in KOH; sterigmata measuring 4.5–9 µm long. Cystidia absent. Pileipellis a cutis made up of hyphae 4–7.5 µm in width, subcylindric, marginally thickened walls (up to 0.5 µm), interwoven to subparallel, pale yellow in KOH; terminal cells 25–47 × 4–8 µm, subclavate to subcylindrical with a rounded-off apex. Clamp connections were not observed in all tissues.

Habitat—Solitary to scattered on the ground of forests dominated by fagaceous trees.

Known distribution—Southern China (Hainan Province).

Additional specimen examined—CHINA. Hainan Province: Wuzhishan, Hainan Tropical Rainforest National Park, elev. 600 m, 9 June 2022, X. Zhang039-1(FHMU7716).

Notes—Our molecular data indicated that the new species *C. albimarginatus* falls into subg. *Imperforati* (Figure 1). Moreover, the morphological features of the new species are also consistent with the concept of the subgenus defined by Cao et al. [10]. Morphologically, *C. albimarginatus* is similar to some other species of subg. *Imperforati*, viz. Chinese *C. badiogriseus* T. Cao & H.S. Yuan, Indian *C. albostrigosus* C.K. Pradeep & K.B. Vrinda, *C. parvogriseus* U. Singh, K. Das & Buyck, and *C. indicus* Deepika, Upadhyay & Reddy, which all share the common features including a small basidioma, a smooth or slightly wrinkled hymenophore, broadly ellipsoid basidiospores, and an absence of clamp connections. However, *C. badiogriseus* has a darker pileus, a smooth, brownish gray to gray hymenophore, larger basidiospores (8–10.5 × 6.8–7.5 µm), and its distribution in temperate China [10]; *C. albostrigosus* has a basidioma with white strigose hairs, larger basidiospores (9–11.5 × 6–8 µm), and its association with trees of Dipterocarpaceae [29]; *C. parvogriseus* has a much paler, white to grayish white hymenophore and larger basidiospores (7–12 × 6.5–9 µm) [40]; *C. indicus* has a light brownish to sand-colored pileus, a yellowish gray stipe, larger basidiospores (7.5–10.5 × 6–7 µm), and its association with trees of Pinaceae [33]. Phylogenetically, these species referred to above formed a well-supported clade (BS = 99%, PP = 1.0) (Figure 1). A BLAST comparison of the *C. albimarginatus* sequences with those available in GenBank revealed that the 28S sequence showed 96.70%, 95.77%, 95.75%, and 95.42% similarity to those of *C. albostrigosus*, *C. badiogriseus*, *C. parvogriseus*, and *C. indicus*, respectively. The highest similarity for the ITS sequence was observed with *C. atratus* (Corner) Yomyart, Watling, Phosri, Piap. & Sihan. (96.39% identity).

***Craterellus involutus*** N.K. Zeng & T. Jiang, **sp. nov.**

MycoBank: MB 856896

**Figure 4:**
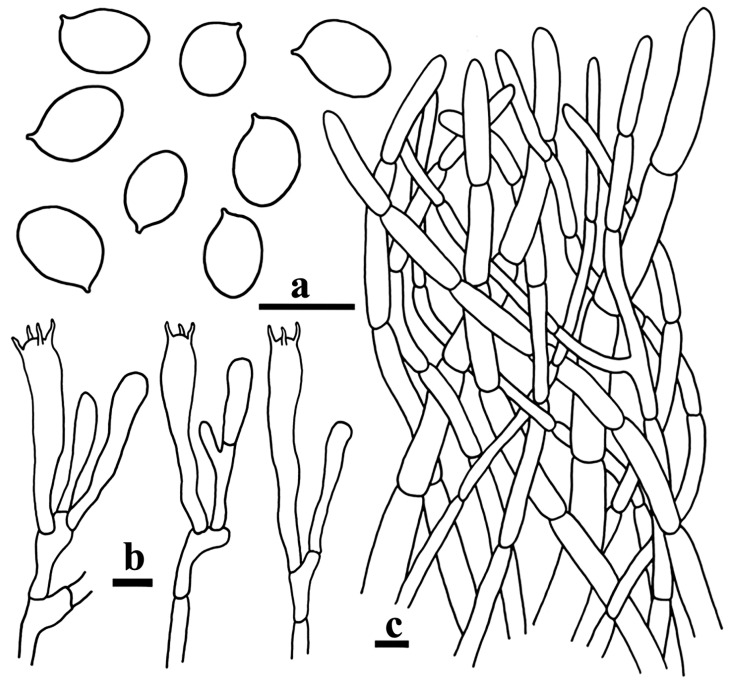
Microscopic structures of *Craterellus involutus* (FHMU7709, holotype). (**a**) Basidiospores. (**b**) Basidia. (**c**) Pileipellis. Scale bars: (**a**–**c**) = 10 µm. Drawing by T. Jiang.

Figure 2c,d and Figure 4.

Etymology—Latin “*involutus*”, meaning curly, refers to the incurved margin of the new species.

Holotype—CHINA. Fujian Province: Sanming Prefecture-level City, Jiangle County, Longqishan National Nature Reserve, elev. 600 m, 21 August 2023, N.K. Zeng8178 (FHMU7709). 28S = PQ604686, *TEF*1 = PQ641589.

Diagnosis—*Craterellus involutus* is differentiated from the closest *Craterellus* species by a basidioma without a distinct boundary between the pileus and stipe, an incurved pileal margin, smaller basidiospores, a trichodermal pileipellis made up of uninflated hyphae, and its connection with Fagaceae trees.

Description—Pileus 1.3–4.7 cm diameter, infundibuliform; margin obviously incurved; surface dry, velutinate, pale brown (4D7) to brownish-black (4F7); context exceedingly thin, grayish-brown (4E7). Hymenophore nearly smooth, with slight rugulose, ashen gray (5C1). Stipe 1.2–5 × 0.4–1.4 cm, axial, hollow, unified with the pileus; dry surface, ashen gray (5C1). Odor not distinctive. Spore print not obtained.

Basidiospores [200/10/3] 8–9.25–10.5(–11.5) × 5–5.95–7 µm, Q = 1.36–1.80(–1.82), Qm = 1.56 ± 0.02, globose to subglobose, sometimes broadly ellipsoid, smooth, marginally thickened walls (up to 0.5 µm), yellowish coloration in KOH. Basidia 50–62 × 6.5–8.5 µm, 2–5-spored, subclavate to subcylindrical, somewhat curving, marginally thickened walls (up to 0.5 µm), faintly yellow in KOH; sterigmata measuring 5.5–8 µm long. Cystidia absent. Pileipellis a trichoderm made up of hyphae 3–11 µm in width, subcylindric, marginally thickened walls (0.5–1.0 µm), yellowish in KOH; terminal cells 35–85 × 5.5–12 µm, subclavate to subcylindrical with a rounded-off apex. Clamp connections were not observed in all tissues.

Habitat—Gregarious on the ground of forests dominated by fagaceous trees.

Known distribution—Southeastern China (Fujian Province).

Additional specimens examined—CHINA. Fujian Province: Sanming Prefecture-level City, Jiangle County, Longqishan National Nature Reserve, elev. 600 m, 21 August 2023, N.K. Zeng8119 (FHMU7708).

Notes—Our molecular data indicated that the new species *C. involutus* is embedded in subg. *Craterellus* (Figure 1). Moreover, the morphological attributes are also in agreement with the concept of the subgenus [10]. Morphologically, *C. involutus* is similar to some other species of subg. *Craterellus*, viz. Chinese *C. croceialbus* T. Cao & H.S. Yuan, *C. macrosporus* T. Cao & H.S. Yuan, and *C. squamatus* T. Cao & H.S. Yuan, European *C. cornucopioides* s.s., North American *C. fallax* A.H. Sm., and *C. calicornucopioides* D. Arora & J.L. Frank, which all share common features including a dark-color pileus, a smooth or wrinkled hymenophore, and an absence of a distinct boundary between the pileus and stipe. However, *C. croceialbus*, *C. macrosporus*, and *C. squamatus* are distributed in temperate China [10]. Moreover, *C. croceialbus* has a pileus that is brownish gray to grayish brown, with an orange-white margin, and larger basidiospores (10–12 × 6.8–8 µm) [10]; *C. macrosporus* has a brownish gray to grayish brown pileus and larger basidiospores (12.8–14.5 × 9–11 µm) [10]; *C. squamatus* has a smaller, light brown to dark brown pileus and larger basidiospores (12–13.8 × 8.5–9.5 µm) [10]. *Craterellus cornucopioides* s.s. has larger basidiospores (13–14 × 7–8 µm) and occurs in temperate regions [51]; *C. fallax* has larger basidiospores (10–13 × 7–9 µm) and is associated with trees of Pinaceae [37]; *C. calicornucopioides* has a larger basidioma (up to 20 cm) with a wavy pileal margin, larger basidiospores (11–14 × 8–10 µm), and an occurrence of numerous clamp connections [31]. Phylogenetically, these species referred to above formed a well-supported clade (BS = 92%, PP = 1.0). The 28S sequence data showed 95.95%, 95.21%, 94.96%, and 94.65% similarity to those of *C. squamatus*, *C. croceialbus*, *C. fallax*, and *C. macrosporus*, respectively, while the *TEF*1 sequence data show 96.46%, 94.8%, and 90.53% similarity to those of *C. squamatus*, *C. fallax*, and *C. croceialbus*, respectively.

*Craterellus cornucopioides* var. *mediosporus* Corner and *C. verrucosus* Massee, two taxa originally described from Malaysia, also exhibit morphological similarities to *C. involutus*. Nevertheless, *C. cornucopioides* var. *mediosporus* has a cutis pileipellis and is distributed in tropical areas [1]; *C. verrucosus* has a pale-color pileus, a thicker hymenophore, and a pileipellis that typically consists of broader hyphae (up to 20 µm) [1].

***Craterellus longitipes*** N.K. Zeng & T. Jiang, **sp. nov.**

MycoBank: MB 856897

**Figure 5:**
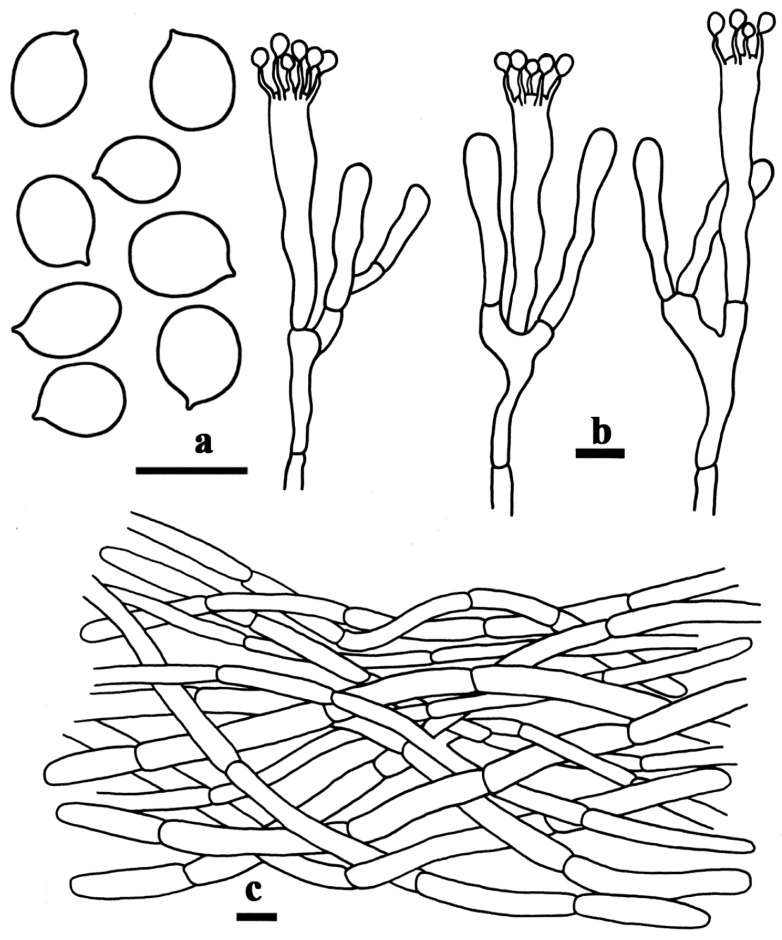
Microscopic structures of *Craterellus longitipes* (FHMU7705, holotype). (**a**) Basidiospores. (**b**) Basidia. (**c**) Pileipellis. Scale bars: (**a**–**c**) = 10 µm. Drawing by T. Jiang.

Figure 2e,f and Figure 5.

**Etymology**—Latin “*longi*-”, meaning long, “*tipes*”, meaning stipe, refer to the long stipe of the new species.

**Holotype**—CHINA. Fujian Province: Sanming Prefecture-level City, Jiangle County, Longqishan National Nature Reserve, elev. 350 m, 21 August 2023, N.K. Zeng8131 (FHMU7705). 28S = PQ604683, ITS = PQ611010, *TEF*1 = PQ641586.

**Diagnosis**—*Craterellus longitipes* is differentiated from its closest relatives in *Craterellus* by a pale brown to brown basidioma, a prominent separation between pileus and stipe, a long stipe, a pileipellis made up of uninflated hyphae, and its relationship with Fagaceae trees.

**Description**—Pileus 2–5.3 cm in diameter, center strongly depressed; margin broadly wavy or unevenly folded; surface dry, slightly rugose, pale brown to brown (4D7); context very thin, blackish-brown (4E7). Hymenophore veined, decurrent; folds 0.05–0.1 cm broad, usually forked, commonly anastomosing, distant, ashen gray (4B2). Stipe 4.2–7 × 0.4–0.7 cm, central, hollow, subcylindrical, frequently flexuous, with prominent separation between pileus and stipe; dry surface, blackish-brown (4F5). Odor not distinctive. Spore print not obtained.

Basidiospores [180/9/3] 6–7.1–8(–9) × 4.5–5.03–5.5(–6) µm, Q = 1.27–1.60(–1.70), Qm = 1.41 ± 0.01, subglobose to ellipsoid or broadly ellipsoid, smooth, marginally thickened walls (up to 0.6 µm), yellowish in KOH. Basidia 36–70 × 4–9 µm, 3–6-spored, subclavate to subcylindrical, marginally thickened walls (up to 0.5 µm), colorless or yellowish in KOH; sterigmata measuring 4.5–10 µm long. Cystidia absent. Pileipellis a cutis made up of hyphae 4.5–8 µm in width, mostly cylindrical, occasionally branched, thin to marginally thickened walls (up to 1 µm), yellowish in KOH; terminal cells 27–74.5 × 7–11.5 µm, subcylindrical or clavate with a rounded-off apex. Clamp connections were not observed in all tissues.

**Habitat**—Gregarious on the ground of forests dominated by fagaceous trees.

**Known distribution**—Southeast China (Fujian Province).

**Additional specimens examined**—CHINA. Fujian Province: Sanming Prefecture-level City, Jiangle County, Longqishan National Nature Reserve, elev. 600 m, 21 August 2023, N.K. Zeng8131-1(FHMU7706); same geographical location and date, N.K. Zeng8131-2 (FHMU7707).

**Notes**—Our molecular data indicated that the subgeneric rank of the new species *C. longitipes* should be further defined (Figure 1). It is worth noting that the closely related taxa of *C. longitipes*, viz. *C. connatus* G.P. Zhao, J.J. Hu, B. Zhang & Y. Li and *C. striatus* G.P. Zhao, J.J. Hu, B. Zhang & Y. Li, both described from northeastern China, are in the same species-level phylogenetic branches with strong support (BS = 100%, PP = 1.0). (Figure 1: lineage 3). By comparing the 28S and *TEF*1 sequences of *C. conatus* and *C. striatus* in GenBank, high identities of 99.89% and 99.74% were observed, respectively. Moreover, the two taxa do not exhibit any major morphological differences [17]. And thus, we are sure that *C. striatus* is synonymous with *C. connatus*. Morphologically, *C. longitipes* is similar to *C. atrobrunneolus* T. Cao & H.S. Yuan and *C. conatus/striatus*; both share the common features including a brown pileus, a gray hymenophore, and an absence of clamp connections. However, *C. atrobrunneolus* has a dark brown to almost black pileus, a shorter stipe, and wider hyphae in pileipellis (up to 11 µm) [15]; *C. conatus/striatus* has a hymenophore sometimes with a strongly anastomosing vein, a shorter stipe, and wider hyphae in pileipellis (up to 16 µm), and its distribution is in temperate areas [17]. Phylogenetically, the 28S sequence data showed 97.63% and 97.10% similarity to those of *C. conatus/striatus* and *C. atrobrunneolus*, respectively, while the ITS sequence showed 98.85% similarity with *C. atrobrunneolus*, and the *TEF*1 sequence showed 97.51% similarity with *C. conatus/striatus*.

## 4. Discussion

With more and more molecular data, numerous previously published taxa have been re-assessed, which have updated the taxonomy of *Craterellus* [2,15,16,17,39]. As an example, the European *C. cornucopioides*, distinguished by its brown to black pileus and smooth hymenophore, was once considered a widely distributed species [1]. However, recent studies have showed that collections named “black trumpet species” represent multiple taxa rather than a single widely distributed species. In China, many specimens labeled as “*C. cornucopioides*” were re-evaluated; several species such as *C. badiogriseus*, *C. croceialbus*, *C. involutus*, *C. parvopullus*, *C. macrosporus*, and *C. squamatus* were described in previous or present studies [10,15,16].

In present study, the species richness of *Craterellus* in China was uncovered, revealing seventeen species-level phylogenetic branches (Figure 1). Three lineages (6, 8, and 14) were classified as previously unrecorded taxa, viz. *C. albimarginatus*, *C. involutus*, and *C. longitipes*; twelve (lineages 1, 3–5, 9–13, 15–17) are classified as previously described species, viz. *C. albidus*, *C. atrobrunneolus*, *C. aureus*, *C. badiogriseus*, *C. croceialbus*, *C. lutescens*, *C. macrosporus*, *C. connatus/striatus*, *C. minor*, *C. parvopullus*, *C. fulviceps*, and *C. squamatus*; and two (lineages 2, 7) remain undescribed due to the paucity of materials. Geographically, *C. badiogriseus*, *C. connatus/striatus*, *C. croceialbus*, *C. macrosporus*, and *C. squamatus* occur in temperate areas of China, and other representatives of the group, viz. *C. albidus*, *C. albimarginatus*, *C. atrobrunneolus*, *C. aureus*, *C. fulviceps*, *C. involutus*, *C. longitipes*, *C. lutescens*, *C. minor*, *C. parvopullus*, and *C. yunnanensis* (W.F. Chiu) Buyck, are from subtropical/tropical China.

In previous studies, six subgenera of *Craterellus* were described [10]. The systematic positions of most Chinese *Craterellus* species have been defined; they are members of subg. *Cariosi*, subg. *Craterellus*, subg. *Imperforati*, subg. *Lamelles*, and subg. *Ovoidei* (Table 2). Besides *C. yunnanensis*, one species being absent of molecular data, the systematic position of *C. atrobrunneolus*, *C. connatus/striatus*, and *C. longitipes* should be further defined (Figure 1).

Key to accepted *Craterellus* taxa in China
1. Absence of obvious demarcation between pileus and stipe21. Presence of obvious demarcation between pileus and stipe82. Pileus colored with vivid yellow to orange*C. aureus*2. Pileus colored with gray brown, brown, dark brown to almost black33. Pileal surface covered with scabrous*C. squamatus*3. Pileal surface subglabrous to glabrous44. Basidiospores average length <8 µm, occurring in tropical regions*C. parvopullus*4. Basidiospores average length >8 µm, occurring in subtropical or temperate regions55. Pileal surface colored with blackish brown, blackish to almost black, basidiospores average length <11 µm.65. Pileal surface colored with brown, gray-brown to dark brown, lacking any black tinge, basidiospores average length >11 µm. 76. Basidia longer (up to 106 µm), pileipellis a cutis, and distributed in temperate regions*C. badiogriseus*6. Basidia shorter (up to 62 µm), pileipellis a trichoderm, and distributed in subtropical regions*C. involutus*7. Margin of pileus colored with dark brown, basidiospores larger measuring 12.8–14.5 × 9–11 µm*C. macrosporus*7. Margin of pileus colored with orange-white, basidiospores smaller measuring 10–12 × 6.8–8 µm*C. croceialbus*8. Basidomata very pale, whitish, grow on dead wood*C. albidus*8. Basidomata brown, yellow, grow on ground99. Hyphal clamp connections absent109. Hyphal clamp connections abundant1310. Hymenophore slightly veined, white tinged with violet, stipe beige-almond, and distributed in tropical regions*C. albimarginatus*10. Hymenophore with well-developed veins, gray, stipe brown, and distributed in subtropical or temperate regions1111. Stipe shorter (up to 3.5 cm), hymenophore with strongly anastomosing vein, and distributed in temperate regions *C. connatus/striatus*11. Stipe longer (up to 5 cm), hymenophore veined, and distributed in subtropical regions1212. Pileus colored with pale brown to brown, pileipellis composed of narrower hyphae (up to 8 µm)*C. longitipes*12. Pileus colored with dark brown to almost black, pileipellis composed of wider hyphae (up to 11 µm)*C. atrobrunneolus*13. Basidiospores shorter (6–7.5 µm), terminal cells wider (up to 19 µm)*C. yunnanensis*13. Basidiospores longer (8–11.5 µm), terminal cells narrower (up to 10 µm)1414. Pileus colored with brown, hymenophore veined, or sometimes smooth*C. lutescens*14. Pileus colored with grayish-yellow to fulvous, hymenophore veined, never smooth1515. Hymenophore colored with yellowish, stipe colored with egg-yolk yellow*C. fulviceps*15. Hymenophore colored with white to pale, stipe colored with pale lemon yellow*C. minor*

## Figures and Tables

**Table 1:** Taxa, vouchers, locations, and GenBank accession numbers of DNA sequences used in this study.

Taxon	Voucher	Locality	GenBank Accession Nos.			References
			28S	ITS	*TEF*1	
*Craterellus albidus*	HGASMF01-3581	China	MT921161	—	—	[28]
*C. albidus*	HGASMF01-10046	China	MT921162	—	—	[28]
***C. albimarginatus***	**ZX039 (FHMU7715) (holotype)**	**China**	**PQ604676**	**PQ611003**	—	**Present study**
***C. albimarginatus***	**ZX039-1 (FHMU7716)**	**China**	—	**PQ611004**	—	**Present study**
*C. albostrigosus*	CAL 1624	India	MG593194	—	—	[29]
*C. atratoides*	TH8243	Guyana	—	KT339209	—	[30]
*C. atratoides*	MCA1313	Guyana	JQ915119	JQ915093	—	[30]
*C. atratoides*	TH9232	Guyana	JQ915137	JQ915111	—	[30]
*C. atratoides*	TH8473	Guyana	JQ915129	JQ915103	—	[30]
*C. atratoides*	AMV1965a	Colombia	KT724157	KT724106	—	Unpublished
*C. atratoides*	AMV1959	Colombia	KT724156	—	—	Unpublished
*C. atratoides*	AMV1870	Colombia	—	KT354698	—	Unpublished
*C. atratoides*	AMV1990	Colombia	—	KT354699	—	Unpublished
*C. atratus*	AMV1832	Colombia	KT724158	KT724107	—	Unpublished
*C. atratus*	TH9203	Guyana	JQ915133	JQ915107	—	[30]
*C. atratus*	MCA990	Guyana	JQ915126	JQ915100	—	[30]
*C. atratus*	MCA1070	Guyana	JQ915118	JQ915092	—	[30]
*C. atrobrunneolus*	Yuan13878	China	MN894058	MN902353	—	[15]
***C. atrobrunneolus***	**N.K. Zeng8189 (FHMU7704)**	**China**	**PQ604677**	**PQ611005**	**PQ641578**	**Present study**
*C. atrocinereus*	Arora15001	United States	—	KR560049	—	[31]
*C. atrocinereus*	JLF3750	United States	—	KR560048	—	[31]
*C. aureus*	N.K. Zeng1057 (FHMU2407)	China	OL439672	OM469019	**PQ641579**	[16]
*C. aureus*	N.K. Zeng3141 (FHMU2102)	China	OL439674	OM469020	**PQ641580**	[16]
*C. badiogriseus*	Yuan 14779	China	MW979533	MW980549	MW999433	[10]
*C. caeruleofuscus*	MH17001	United States	MT237468	MH558300	—	[15]
*C. calicornucopioides*	JLF3744	United States	—	KR560046	—	[31]
*C. calicornucopioides*	Arora 15002	United States	—	KR560047	—	[31]
*C. calyculus*	Mushroom Observer # 321697	United States	—	MK607596	—	Unpublished
*C. carolinensis*	FLAS-F-59997	United States	—	KY654712	—	[32]
*C. cinereofimbriatus*	TH9264	Guyana	JQ915138	JQ915112	—	[30]
*C. cinereofimbriatus*	TH9075	Guyana	JQ915131	JQ915105	—	[30]
*C. cinereus*	107-08	India	JF412276	JF412278	—	[33]
*C. cinereus*	AST2015	Pakistan	—	MF374488	—	[34]
*C. cornucopioides*	HbO-53302	Norway	AF105301	—	—	[2]
*C. cornucopioides*	UPSF-11792	Sweden	AF105297	—	—	[2]
*C. cornucopioides*	WA0000071019	Poland	—	MK028881	—	[35]
*C. cornucopioides*	—	China	AJ279572	—	—	[12]
*C. cornucopioides*	CNF 1/7292	Croatia	—	MK169230	—	[36]
*C. croceialbus*	Yuan 14623	China	MW979529	MW980572	MW999430	[10]
*C. croceialbus*	Yuan 14647	China	MW979530	MW980573	MW999431	[10]
*C. cornucopioides* var. *mediosporus*	268-06	India	JF412275	JF412277	—	[33]
*C. connatus*	HMJAU 61462 (T)	China	OM509448	—	ON125915	[17]
*C. connatus*	HMJAU 61462	China	—	—	ON125916	[17]
*C. excelsus*	TH8235	Guyana	JQ915128	JQ915102	—	[30]
*C. excelsus*	TH7515	Guyana	JQ915127	JQ915101	—	[30]
*C. fallax*	PBM3290	United States	—	GU590923	—	[37]
*C. fulviceps*	MHHNU10567 (FHMU6553)	Hunan, central China	OL439678	OL439548	—	[16]
***C. fulviceps***	**N.K. Zeng7833 (FHMU7710)**	**China**	**PQ604678**	**PQ611006**	**PQ641581**	**Present study**
***C. fulviceps***	**N.K. Zeng7833-1 (FHMU7711)**	**China**	**PQ604679**	**PQ611007**	**PQ641582**	**Present study**
***C. fulviceps***	**N.K. Zeng7940 (FHMU7712)**	**China**	**PQ604680**	**—**	**PQ641583**	**Present study**
***C. fulviceps***	**N.K. Zeng8033 (FHMU7713)**	**China**	**PQ604681**	**PQ611008**	**PQ641584**	**Present study**
***C. fulviceps***	**N.K. Zeng8163 (FHMU7714)**	**China**	**PQ604682**	**PQ611009**	**PQ641585**	**Present study**
*C. ignicolor*	UPSF-11794	United States	AF105314	—	—	[2]
*C. indicus*	PUN3884	India	HM113529	HM113530	—	[33]
*C. indicus*	MSR6	India	—	HQ450769	—	[33]
*C. inusitatus*	CAL 1625	India	MG593195	—	—	[29]
***C. involutus***	**N.K. Zeng8119 (FHMU7708)**	**China**	**PQ604685**	—	**PQ641588**	**Present study**
***C. involutus***	**N.K. Zeng8178 (FHMU7709) (holotype)**	**China**	**PQ604686**	—	**PQ641589**	**Present study**
***C. longitipes***	**N.K. Zeng8131 (FHMU7705) (holotype)**	**China**	**PQ604683**	**PQ611010**	**PQ641586**	**Present study**
***C. longitipes***	**N.K. Zeng8131-1 (FHMU7706)**	**China**	—	**PQ611011**	**PQ641587**	**Present study**
***C. longitipes***	**N.K. Zeng8131-2 (FHMU7707)**	**China**	**PQ604684**	**PQ611012**	—	**Present study**
*C. lutescens*	ma023	Italy	MN592820	MN595294	—	[38]
*C. lutescens*	L.P. Tang1647 (FHMU6547)	China	OL439679	OL439549	—	[16]
*C. lutescens*	W.H. Zhang441-1 (FHMU6544)	China	OL439681	OL439550	—	[16]
*C. luteus*	GDGM46432	China	MG727898	MG727897	—	[11]
*C. luteus*	GDGM48105	China	MG701171	MG727896	—	[11]
*C. macrosporus*	Yuan 14782	China	MW979531	MW979531	—	[10]
*C. melanoxeros*	SS576	Sweden	JQ976983	—	—	[39]
*C. minor*	420526MF0891	China	MG712381	—	—	Unpublished
*C. minor*	MHHNU32505 (FHMU6554)	China	OL439684	OL439553	**PQ641597**	[16]
*C. odoratus*	14026h2	United States	MN227279	—	—	Unpublished
*C. odoratus*	14026h1	United States	MN227278	—	—	Unpublished
*C. odoratus*	UPSF-11799	United States	AF105306	—	—	[2]
*C. olivaceoluteus*	TH9205	Guyana	JQ915135	JQ915109	—	[30]
*C. olivaceoluteus*	MCA3186	Guyana	JQ915124	JQ915098	—	[30]
*C. parvogriseus*	CAL1533	India	MF421098	MF421099	—	[40]
*C. parvogriseus*	KNPS_WC18158	Korea	MT974136	—	—	[41]
*C. parvopullus*	N.K. Zeng4913 (FHMU6555)	China	OL439685	OM334829	**PQ641592**	[16]
*C. parvopullus*	N.K. Zeng4912 (FHMU6556)	China	OL439686	OM334828	**PQ641590**	[16]
*C. parvopullus*	N.K. Zeng4911 (FHMU6557)	China	OL439687	OM334827	**PQ641591**	[16]
***C. parvopullus***	**N.K. Zeng7557 (FHMU7717)**	**China**	**PQ604687**	—	**PQ641593**	**Present study**
***C. parvopullus***	**N. K. Zeng7521 (FHMU7718)**	**China**	**PQ604688**	—	**PQ641594**	**Present study**
***C. parvopullus***	**N. K. Zeng7512 (FHMU7719)**	**China**	**PQ604689**	—	—	**Present study**
*C. pleurotoides*	MCA3124	Guyana	JQ915123	JQ915097	—	[30]
*C. pleurotoides*	TH9220	Guyana	JQ915136	JQ915110	—	[30]
*C. shoreae*	CAL_F_1396	India	KY290585	—	—	[15]
*C. sinuosus*	TF1802	United States	U87992	—	—	[42]
*Craterellus* sp.	MHHNU32154 (FHMU6552)	China	OL439677	OL439547	**PQ641595**	[16]
***Craterellus* sp.**	**N.K. Zeng8042 (FHMU7703)**	**China**	**PQ604690**	**PQ611013**	**PQ641596**	**Present study**
*C. squamatus*	Yuan 14520	China	MW979534	MW980571	MW999434	[10]
*C. squamatus*	Yuan 14721	China	MW979535	MW980570	MW999435	[10]
*C. strigosus*	TH9204	Guyana	JQ915134	JQ915108	—	[30]
*C. strigosus*	MCA1750	Guyana	JQ915120	JQ915094	—	[30]
*C. striatus*	HMJAU 61463 (T)	China	OM509446	—	ON125913	[17]
*C. striatus*	HMJAU 61463	China	OM509447	—	ON125914	[17]
*C. tubaeformis*	DAVFP26257	Canada	—	HM468491	—	[43]
*C. tubaeformis*	UPS-11797	United States	AF105311	—	—	[2]
*C. tubaeformis*	TRTC52516	Belgium	—	HM468496	—	[43]
*C. tubaeformis*	UPSF-11793	Sweden	AF105307	—	—	[2]
*C. tubaeformis*	BB 07.293	Slovakia	KF294640	—	—	[44]
*C. tubaeformis*	TRTC52235	Belgium	—	HM468497	—	[43]
*C. tubaeformis*	BR089347	Canada	—	HM468493	—	[43]
*C. tubaeformis*	OSC-41280	United States	AF105313	—	—	[2]
*C. tubaeformis*	GCB1905	Belgium	—	MT004784	—	[2]
*C. tubaeformis*	UPSF-11795	United States	AF105308	—	—	[2]
*Hydnum minus*	N.K. Zeng2819 (FHMU2461)	China	KY407528	KY407533	KY407534	[25]
*H. cremeoalbum*	N.K. Zeng2511 (FHMU2153)	China	KY407527	KY407532	KY407535	[25]

Bold GenBank numbers highlight the newly generated sequences.

**Table 2:** Subgenera and accepted species of *Craterellus* in China.

Subgenus	Species	Locality	Reference
*Cariosi*	*C. lutescens*	Europe	[16]
*Craterellus*	*C. croceialbus*	Liaoning NE China	[10]
	*C. involutus*	Fujian, SE China	Present study
	*C. macrosporus*	Liaoning NE China	[10]
	*C. squamatus*	Liaoning NE China	[10]
*Imperforati*	*C. albidus*	Guizhou, SW China	[28]
	*C. albimarginatus*	Hainan, southern China	Present study
	*C. badiogriseus*	Liaoning, NE China	[10]
*Lamelles*	*C. fulviceps*	Hunan, central China	[16]
	*C. minor*	Hunan, central China	[16]
*Ovoidei*	*C. aureus*	Hong Kong, southern China	[16]
	*C. parvopullus*	Hainan, southern China	[16]
—	*C. atrobrunneolus*	Yunnan, SW China	[15]
	*C. connatus/striatus*	Liaoning, NE China	[17]
	*C. longitipes*	Fujian, SE China	Present study
	*C. yunnanensis*	Yunnan, SW China	[52]

SW: southwestern, NE: northeastern, SE: southeastern.

## Data Availability

The datasets presented in this study can be found in online accession number(s), which are as follows: National Center for Biotechnology Information (NCBI) GenBank, https://www.ncbi.nlm.nih.gov/genbank/, PQ604676-PQ604690, PQ611003-PQ611013, PQ641578-PQ641597 and MycoBank, https://www.mycobank.org/, MB856885, MB856896, MB856897.
